# Testing Behavior Change Techniques to Encourage Primary Care Physicians to Access Cancer Screening Audit and Feedback Reports: Protocol for a Factorial Randomized Experiment of Email Content

**DOI:** 10.2196/resprot.9090

**Published:** 2018-02-16

**Authors:** Gratianne Vaisson, Holly O Witteman, Zachary Bouck, Caroline A Bravo, Laura Desveaux, Diego Llovet, Justin Presseau, Marianne Saragosa, Monica Taljaard, Shama Umar, Jeremy M Grimshaw, Jill Tinmouth, Noah M Ivers

**Affiliations:** ^1^ Department of Epidemiology Faculty of Medicine Laval University Quebec City, QC Canada; ^2^ Office of Education and Continuing Professional Development Faculty of Medicine Laval University Quebec City, QC Canada; ^3^ Department of Family and Emergency Medicine Faculty of Medicine Laval University Quebec City, QC Canada; ^4^ Research Centre of the Centre Hospitalier Universitaire de Québec Laval University Quebec City, QC Canada; ^5^ Institute for Health Systems Solutions and Virtual Care Women’s College Hospital Toronto, ON Canada; ^6^ Prevention and Cancer Control Cancer Care Ontario Toronto, ON Canada; ^7^ Institute of Health Policy, Management and Evaluation University of Toronto Toronto, ON Canada; ^8^ Clinical Epidemiology Program Ottawa Hospital Research Institute Ottawa, ON Canada; ^9^ School of Epidemiology and Public Health University of Ottawa Ottawa, ON Canada; ^10^ School of Psychology University of Ottawa Ottawa, ON Canada; ^11^ Family Practice Health Centre Women’s College Hospital Toronto, ON Canada; ^12^ Department of Medicine University of Ottawa Ottawa, ON Canada; ^13^ Institute for Clinical Evaluative Sciences Toronto, ON Canada; ^14^ Faculty of Medicine University of Toronto Toronto, ON Canada; ^15^ Sunnybrook Research Institute Toronto, ON Canada; ^16^ Department of Family and Community Medicine University of Toronto Toronto, ON Canada

**Keywords:** early detection of cancer, primary health care, feedback, electronic mail, persuasive communication, clinical trials as topic, behavior change techniques, process evaluation, implementation science

## Abstract

**Background:**

Cancer Care Ontario’s Screening Activity Report (SAR) is an online audit and feedback tool designed to help primary care physicians in Ontario, Canada, identify patients who are overdue for cancer screening or have abnormal results requiring follow-up. Use of the SAR is associated with increased screening rates. To encourage SAR use, Cancer Care Ontario sends monthly emails to registered primary care physicians announcing that updated data are available. However, analytics reveal that 50% of email recipients do not open the email and less than 7% click the embedded link to log in to their report.

**Objective:**

The goal of the study is to determine whether rewritten emails result in increased log-ins. This manuscript describes how different user- and theory-informed messages intended to improve the impact of the monthly emails will be experimentally tested and how a process evaluation will explore why and how any effects observed were (or were not) achieved.

**Methods:**

A user-centered approach was used to rewrite the content of the monthly email, including messages operationalizing 3 behavior change techniques: anticipated regret, material incentive (behavior), and problem solving. A pragmatic, 2x2x2 factorial experiment within a multiphase optimization strategy will test the redesigned emails with an embedded qualitative process evaluation to understand how and why the emails may or may not have worked. Trial outcomes will be ascertained using routinely collected administrative data. Physicians will be recruited for semistructured interviews using convenience and snowball sampling.

**Results:**

As of April 2017, 5576 primary care physicians across the province of Ontario, Canada, had voluntarily registered for the SAR, and in so doing, signed up to receive the monthly email updates. From May to August 2017 participants received the redesigned monthly emails with content specific to their allocated experimental condition prompting use of the SAR. We have not yet begun analyses.

**Conclusions:**

This study will inform how to communicate effectively with primary care providers by email and identify which behavior change techniques tested are most effective at encouraging engagement with an audit and feedback report.

**Trial Registration:**

ClinicalTrials.gov NCT03124316; https://clinicaltrials.gov/ct2/show/NCT03124316 (Archived by WebCite at http://www.webcitation.org/6w2MqDWGu)

## Introduction

Health care provider behavior is an important determinant of patients’ use of screening services [1-5]. A number of knowledge translation strategies intended to target provider behavior already exist **,** including audit and feedback [6], reminder/recall systems [7], and incentives [8]. The effectiveness of these strategies varies considerably and may be partly explained by variation in the features of the interventions [6,7], the differing clinical contexts in which the interventions are used, and the extent to which clinicians actually engage with the interventions [9,10]. For example, physicians may not access feedback reports regularly if they lack trust in data quality, if they are not motivated to improve on the indicators measured, or if they encounter organizational or other constraints that interfere with quality improvement [11].

In 2012, the global burden of cancer amounted to 8.2 million deaths, representing 13% of all deaths globally [12,13]. Screening can reduce cancer-related mortality [14] if appropriate tests are used [15] and if a sufficient number of patients from the target population participate [16]. Despite the availability of organized screening programs for colon, breast, and cervical cancer in Ontario, Canada [17], more than one-third of the eligible population in the province are not up to date with screening tests for these cancers [18].

Cancer Care Ontario (CCO) is the agency that oversees population-based cancer screening programs in Ontario. It currently uses a multifaceted strategy to increase cancer screening rates, including public media campaigns, letters mailed to patients overdue for screening, and an audit and feedback tool available to primary care physicians. The online audit and feedback tool, known as the Screening Activity Report (SAR), is updated monthly to help primary care providers identify specific patients who are overdue for screening and/or who have screening results that require follow-up in addition to comparing their performance to the regional average. Use of the SAR by primary care physicians is associated with higher rates of colorectal, breast and cervical screening [19]. However, most primary care physicians in the province do not regularly use the SAR. To encourage use, CCO sends a monthly email to primary care physicians who have registered for the SAR (see Multimedia Appendix 1); however, internal CCO data extracted in 2016 revealed that 50% of email recipients did not open the email, and less than 7% of recipients clicked the enclosed link to log in to their SAR. Therefore, we worked with CCO to develop a study to test theory- and user-informed content to improve the salience and impact of emails, with the objective of increasing SAR access and, ultimately, aligning cancer screening rates with guidelines. In this manuscript, we describe the design of a pragmatic, factorial randomized experiment using the Multiphase Optimization Strategy (MOST) [20] to evaluate the impact of different components in the monthly SAR delivered by email to primary care physicians across Ontario and an accompanying process evaluation.

## Methods

### Intervention Design: Monthly Emails Prompting Use of the Screening Activity Report

The team first employed a user-centered design approach informed by principles of behavior change theory to rewrite the monthly emails and identify email components, or behavior change techniques (BCTs), to test in an experiment . BCTs are defined as “active components of an intervention designed to change behavior” [21]. We identified BCTs from the Behavior Change Technique Taxonomy (v1) [22] that could influence primary care physician behavior [23].

The rewriting process emphasized content development and involved focus groups with adopters (physicians who already access and use the SAR) and nonadopters (physicians who have not accessed the SAR for at least 1 year). CB and DL, both qualitative experts at Cancer Care Ontario, led the groups of discussion. Through this process, we identified 3 modifiable components that could be prioritized for testing in a trial. Specifically, we hypothesized that the following BCTs could help increase the effectiveness of the emails: anticipated regret (ie, induce or raise awareness of expectations of future regret about not logging in to the SAR), material incentive (behavior) (ie, Identify opportunity for using SAR to access available monetary bonus for achieving high cancer screening rates ), and problem solving (ie, generate or select strategies that include overcoming barriers and/or increasing facilitators to accessing the SAR) (see Multimedia Appendix 2). The details of this process and the iterative changes made based on adopter input will be reported separately.

The BCTs tested are presumed to target 1 or more determinants of behavior (ie, the potential mechanism of action) as described in the theoretical domains framework (Table 1).

**Figure 1:**
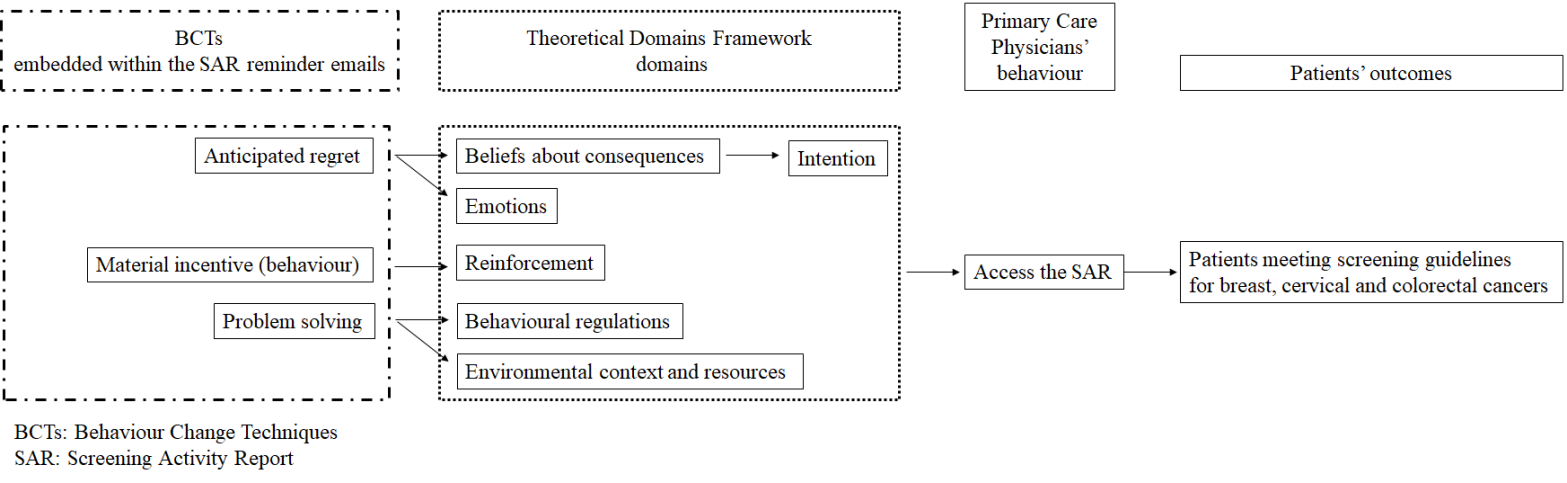
Logic model of the content and hypothesised mechanism of change.

We verified the importance of the selected BCTs with adopters and nonadopters. We used the theoretical domains framework terminology to describe the potentially relevant determinants because mapping between BCTs and theoretical domains framework is already established [21,24]. For example, “anticipated regret” targets emotion and beliefs about consequences, which may drive primary care physicians to access their SAR (Figure 1).

### Study Design

**Figure 2:**
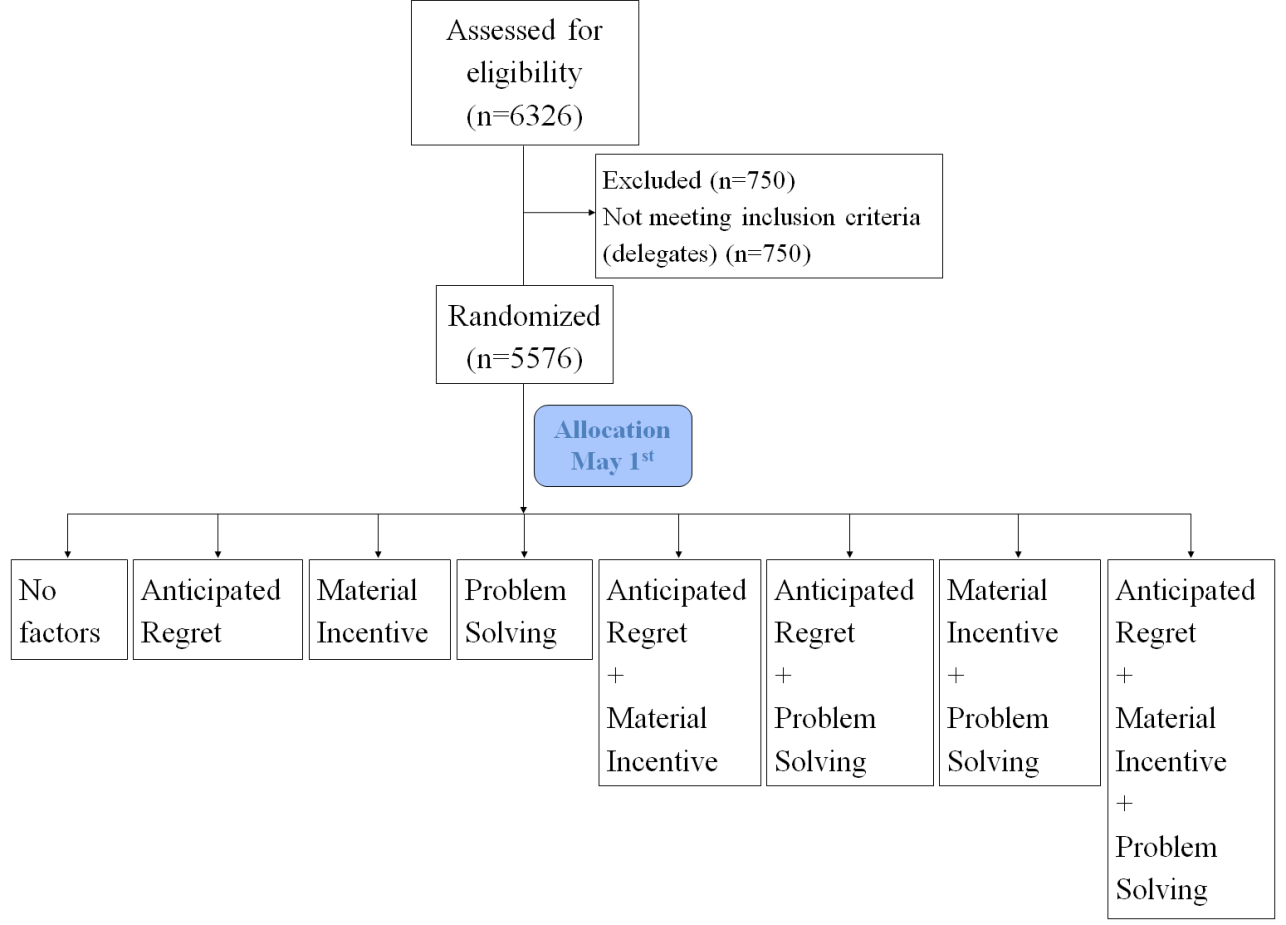
Allocation flow diagram.

This approach follows the MOST framework to optimize and evaluate multicomponent behavioral interventions [20,25]. Factorial experiments allow for the estimation of main effects of multiple factors in a single experiment by combining experimental conditions [26,27]. Thus, such designs are useful options in health behavior research to efficiently compare more than 1 intervention, particularly, as in our case, when there is no expectation that the factors being tested will substantially interact [28,29]. The trial itself is a 2×2×2 factorial randomized experiment with 8 experimental conditions (Figure 2). 

**Table 1:** Content and hypothesized mechanism of change for each behavior change technique.

Items	Behavior change techniques/factors tested		
	Material incentive (behavior)	Anticipated regret	Problem solving
General description^a^	Explicitly link SAR^b^ use to a monetary bonus^c^ (awarded only if achieving high cancer screening rates)	Induce or raise awareness of expectations of future regret about not logging in to the SAR	Provide strategies that aim to overcome identified barriers to accessing the SAR
Operationalization	Logging into the SAR can help you maximize your screening rates and save time when calculating your preventive care bonus.	How would you feel if a patient had a poor outcome because you missed an abnormal test result?	We know accessing the SAR involves work for you and your staff. Here are 3 tips from other Ontario primary care doctors on how to fit using the SAR into your schedule: Email ONE ID at ONEIDBusinessSupport@ehealthontario.on.ca to register a delegate with eHealth Ontario so they can check your report Book calendar time right now to check your report Tackle a few patients at a time
Theoretical domains framework-based determinants	Reinforcement, intention	Emotions, beliefs about consequences, intention	Behavioral regulation, environmental context and resources

^a^The descriptions of the behavior change techniques are in line with the Behavior Change Techniques Taxonomy (v1) [22].

^b^SAR: Screening Activity Report.

^c^The financial incentive was already available from the Ministry of Health for achieving screening thresholds; the communication emphasizes how use of the SAR can help in attaining the bonus funds associated with these thresholds.

Three components in the redesigned SAR email will be tested. Each of these 3 factors in the trial will either be turned on or turned off in the email (Table 2).

The trial received approval from the Research Ethics Board at Women’s College Hospital and is registered on ClinicalTrials.gov [NCT03124316].

### Setting

The setting for this study will be primary care because this is the main point of entry to the health care system for Ontarians. In Ontario, the vast majority of primary care is delivered by primary care physicians, and patient visits to physicians are covered under the provincial health plan. According to Statistics Canada, 92.5% of Ontarians aged 12 years and over have a regular medical doctor, usually a primary care physician [30]. Almost three-quarters (73%) of Ontarians are associated with a primary care physician working in a physician enrollment model, meaning the primary care physician has an identifiable list of patients [31]. Primary care physicians working in physician enrollment models can register for the SAR and thus automatically receive the monthly email update from CCO.

### Participants and Recruitment

Eligible participants will be primary care physicians already registered to access the SAR and receive monthly emails from CCO. In Ontario, as of May 1, 2017, 8462 primary care physicians were working in a patient enrollment model. Of these, 5576 eligible physicians had signed up for the SAR. These 5576 physicians will be automatically enrolled in our study. We received a waiver of consent for providers’ participation because the intervention (email receipt) was considered to pose minimal risk (as primary care physicians were already receiving an email), individual primary care physician recruitment was not feasible, and there were concerns that informing providers about the trial might prime participants to act in a certain manner. Participating physicians can opt out of receiving the SAR emails as usual.

### Allocation and Blinding

A deidentified list of eligible participants will be exported from CCO to an independent statistician who will allocate participants to receive 1 of the 8 experimental conditions via simple randomization using computer-generated random numbers.

The different email versions (featuring the different BCTs to be tested) (see Multimedia Appendix 3) will be sent out in 45-minute intervals starting from 9 AM on the 10th day of each month (or if the day falls on the weekend, on the next business day) for 4 months between May and August 2017. The emails could not be sent at the same time since the Cancer Care Ontario’s outgoing mail server could not handle sending out all of the emails at the same time. That is why a delivery schedule made up of 9 intervals (each 45 minutes long) was devised. To address the concern raised regarding sending time, we randomized the order in which the email versions would be sent (ie, 1 version sent per interval) per month. Delivery order will be determined via block randomization. 

**Table 2:** Experimental conditions. + refers to the presence of the factor and – refers to the absence of the factor.

Experimental condition	Factor 1: anticipated regret	Factor 2: material incentive	Factor 3: problem solving
1	–	–	–
2	+	–	–
3	–	+	–
4	–	–	+
5	+	+	–
6	+	–	+
7	–	+	+
8	+	+	+

All randomization procedures will be conducted in SAS version 9.4 (SAS Institute Inc). Treatment allocation will be concealed from the research team and CCO collaborators. It is not possible to blind participants as they must receive the intervention and may notice differences in email content and design; however, they are unlikely to be aware of the different components of the email being tested.

### Data Collection and Outcomes

In this experiment, all data will be routinely collected from CCO administrative databases. Table 3 details the variables to be collected and used in analysis.

All outcomes will be measured at the level of the physician-participant. The primary outcome will be a dichotomous variable indicating whether or not the primary care physician accessed the SAR at least once between May and August 2017. Secondary outcomes measured at the end of the 4-month trial via CCO database sources will be the total number of times the SAR was accessed (between May and August 2017) and physician adherence to screening guidelines for breast, colon, and cervical cancer for their practice (as of August 31). Process measures will include the number of participants opting out of emails or with emails that bounce back (ie, inactive email address) and the number of calls to CCO and/or eHealth Ontario contact center during the trial regarding the SAR (eg, password retrieval queries).

### Statistical Analyses

Physician baseline characteristics (sex, years of practice), their history of SAR use (previous use/no previous use), their baseline cancer screening rates (breast, cervical, and colon), and their practice characteristics (size of group, rurality, payment model) will be described across experimental conditions.

The unit of analysis for all outcomes will be the primary care physician. The effect of each intervention factor on the primary outcome (accessing the SAR at least once during the 4-month trial) will be analyzed using logistic regression. Indicator variables will be specified for each of the experimental factors using effect coding where +1 indicates the presence and –1 the absence of each factor. With effect coding, the regression coefficient represents the difference in the mean response across all experimental conditions with that factor turned on against the mean for all experimental conditions with that factor turned off. For example, the regression coefficient for “anticipated regret” will represent the difference in the log-odds of accessing the SAR for providers allocated to conditions where this factor is turned on (study arms 2+5+6+8) versus providers allocated conditions where the factor is turned off (study arms 1+3+4+7) (Table 2). The effect of each intervention factor will be expressed as an odds ratio with 95% confidence interval. The model will adjust for history of SAR use as a fixed covariate.

Dichotomous secondary outcomes (participants opting out of emails or with emails that bounce back) will be analyzed as described for the primary outcome. Total number of times the SAR was accessed and number of calls to CCO will be analyzed using Poisson regression with the natural log of the number of person-months as an offset term. For these models, the effect of each intervention factor will be expressed as a rate ratio with 95% confidence interval. In the event of significant overdispersion as assessed by a Lagrange multiplier test, a negative binomial distribution will be specified. For physician adherence to cancer screening guidelines, the dependent variable will be the number of eligible patients who meet a set of cancer-specific guidelines out of the total number of eligible patients listed to that provider and analyzed using logistic regression.

We had no a priori hypotheses about the interactions between the BCTs tested (anticipated regret, material incentive [behavior], and problem solving). Secondary analyses will include all 2-way interaction terms between experimental factors as well as comparison across the 8 experimental conditions. Secondary analyses will examine differences across subgroups defined by physician characteristics (eg, sex, years in practice, baseline screening rates, practice size, history of SAR use) and practice characteristics (eg, size of group, rurality, payment model). The length of the emails will also be explored as a potential effect modifier.

All analyses will be conducted in SAS version 9.4 (SAS Institute Inc) with statistical significance assessed at the 5% level.

### Power Calculation

In this experiment, sample size is predetermined by the number of eligible physicians signed up for the SAR (5576 eligible physicians at the time of randomization). 

**Table 3:** Data collection.

Variable		Definition
**Primary outcome**		
	SAR^a^ access	Whether or not eligible physicians accessed the SAR at least once during the 4-month trial (May to August 2017)
**Secondary outcomes**		
	Number of times SAR accessed	A count of the number of times the physician logged in to the SAR in the 4-month period (May to August 2017) prior to the data cutoff date. Multiple log-ins within a single day will only be counted as 1 log-in.
	Adherence to cancer screening: breast (posttrial)	Proportion of enrolled, eligible patients who are up to date with breast screening at the physician level Numerator: screen-eligible, enrolled patients who had a mammogram in the 24 months before the report cut off date per PEM^b^ physician Denominator: breast screening–eligible, enrolled patients per PEM physician
	Adherence to cancer screening: cervical	Proportion of enrolled, eligible patients who are up to date with cervical screening at the physician level Numerator: screen-eligible, enrolled patients who had a Pap test in the 36 months before the report cutoff date per PEM physician Denominator: Cervical screening–eligible, enrolled patients per PEM physician
	Adherence to cancer screening: colon	Proportion of enrolled, eligible patients who are up to date with colorectal tests intended for screening at the physician level Numerator: screen-eligible, enrolled patients who had either a colonoscopy in the 120 months and/or flexible sigmoidoscopy in the 60 months and/or FOBT^c^ in the 24 months before the report cutoff date per PEM physician Denominator: screen-eligible, enrolled patients per PEM physician
**Process measures**		
	Number of calls to CCO^d^	Number of calls to CCO contact center regarding the SAR (May-August)
	Number of calls to ehealth Ontario	Number of calls to ehealth Ontario contact center regarding a ONEID-related issue (May-August)
	Number of participants opting out	Proportion of participants deciding to opt out of receiving the email during the 4-month trial
	Number of participants with emails that bounced back	Proportion of participants with emails that bounced back (ie, inactive email address)
**Subgroup analyses**		
	History of SAR use	PEM physicians who have never logged in, those who have not logged in for 1 year but did previously, and those with 1 or >1 log-ins in the year prior to the trial
	Baseline cancer screening rate: breast (pretrial)	Proportion of enrolled, eligible patients who are up to date with breast screening at the physician level as of March 31, 2017
	Baseline cancer screening rate: cervical (pretrial)	Proportion of enrolled, eligible patients who are up to date with cervical screening at the physician level as of March 31, 2017
	Baseline cancer screening rate: colon (pretrial)	Proportion of enrolled, eligible patients who are up to date with colorectal tests intended for screening at the physician level as of March 31, 2017
	Sex	Sex of physician
	Years in practice	From the year of graduation
	Practice size	Number of patients enrolled to the physician’s practice
	Size of group	The total number of physicians belonging to the group and practicing within the LHIN^e^ Note: physicians who are part of the physician enrollment model group but practicing in another LHIN will not be included in this count
	Payment model	Identifies the specific type (ie, fee-for-service or capitation)
	Rurality	According to postal code of the PEM physician’s primary practice

^a^SAR: Screening Activity Report.

^b^PEM: physician enrollment model.

^c^FOBT: fecal occult blood test.

^d^CCO: Cancer Care Ontario.

^e^LHIN: Local Health Integration Network.

For testing each experimental factor, a sample size of 5576 physicians achieves 95% power to detect an absolute difference of 4% in SAR use (between those with the factor present versus those with the factor absent) using a 2-sided test at the 5% level of significance assuming a control arm proportion of 0.20 (estimated based on prior CCO data). We would consider small differences in proportions of primary care providers accessing the SAR to be clinically important. For example, if an intervention component resulted in only 4% more primary care providers accessing the SAR (ie, 223 more primary care providers) and if each of those primary care providers identified only a few patients overdue for screening, this would result in cancer screening for an additional 500 to 600 Ontarians.

Screening 500 to 600 more people in over 4 months could be expected to lead to cancer diagnosis and appropriate treatment in less than 1 patient [32]. This positive potential outcome should be placed in context, as screening also has potential harms including false positives and complications at a rate dependent on the nature of the test and the risk of the underlying population. For example, if 1000 people are screened for colon cancer, 1 to 2 people will get extra years of life and 35 people will be falsely diagnosed when taking using fecal occult blood test while 2 people will get extra years of life, 5 people will have cancer prevented and less than 1 person will have complications when using flexible sigmoidoscopy [33]. Patients and family physicians should consider the balance of risks and benefits when considering a given screening test, especially as people age [34]. False positives and overdiagnosis can lead to overtreatment and may have negative psychological impacts on patients [35].

### Embedded Process Evaluation

The process evaluation aims to complement the results of the factorial randomized experiment by exploring how and why the email interventions may (or may not) have resulted in changes in accessing and using the SAR [36]. In semistructured interviews (see Multimedia Appendix 4) with primary care physicians, we will explore how the operationalized BCTs target (or fail to target) determinants of their SAR access (Figure 1).

#### Recruitment

Invitation emails will be sent to primary care physicians participating in the experiment, and a Can $150 (US $121) gift card will be offered as an honorarium. Purposive sampling will be used to ensure both primary care physicians who have not logged in to the SAR and those who have logged at least once are included in the sample. We will use convenience sampling (physicians within study investigators’ professional and personal networks) and snowball sampling (asking participants to forward the recruitment email to potential participants in their own networks of physician colleagues). We will seek recruitment of both SAR users and those who have not used the SAR, with sampling continuing until saturation. To the greatest extent possible within feasibility constraints, we will attempt to recruit physicians exposed to each of the 3 factors during the trial. Interviews and recruitment will continue until no new themes emerge and saturation is apparent regarding the information given for both groups of physicians (those who have and have not accessed the SAR).

#### Data Collection

Telephone-based semistructured interviews will be conducted with eligible primary care physicians who received a rewritten email as part of the trial. It is anticipated that interviews will last about 30 minutes. All interviews will be audio-recorded. If participants are unable to retrieve an email they received, we will send them the email with all operationalized BCTs immediately prior to the interview. The interview guide will be pilot-tested prior to use and refined as needed throughout the interview process (see Multimedia Appendix 4). The guide includes specific questions developed for each physician group (ie, those who accessed the SAR and those who did not access the SAR at least once during the trial). The questions are also tailored to the email they received to inform about the potential mechanisms of action by which the included BCTs produced change or not regarding SAR access and use [21]. The interview will also explore general impressions of the redesigned emails and about the SAR more generally.

#### Data Analysis

All audio-recorded interviews will be transcribed. Qualitative data will be analyzed using the framework method which involves the organization and summary of qualitative data by both cases (rows) and themes (columns) [37]. Included BCTs and targeted determinants corresponding to the theoretical domains framework will be applied as deductive codes (beliefs about consequences, emotions, intention, reinforcement, and behavioral regulation). A second level of inductive coding will capture mechanisms of action and will seek to link these mechanisms to specific intervention components (BCTs) whenever possible. Open coding will be applied when emerging themes do not align with theoretical domains framework constructs or when participants highlight the intervention’s failure to activate a potential mechanism of action. All data will be coded by 2 independent analysts using NVivo (QSR International Pty Ltd) (GV and 1 member of the research team). A third analyst will be consulted if discrepancies arise and no consensus can be reached. Once common themes are established across all interviews, the analytical framework will be applied and data will be charted in the framework matrix [37]. We will continue interviews until no new themes emerge and then conduct 3 additional interviews to verify saturation of themes.

Quantitative data from the trial (including exploratory subgroup analyses) and qualitative data from the pretrial focus groups and the posttrial semistructured interviews will be integrated and interpreted by 2 members of the team. In keeping with recommendations for triangulation [38,39], findings regarding barriers and facilitators of logging in and using the SAR and regarding primary and secondary trial outcomes will be summarized in 1 table, known as a convergence coding matrix. Convergence of findings across all data sets will be assessed: full agreement (data convergence), partial agreement (complementarity between data), conflicting findings (discord) or silence (finding identified in only 1 data source and no additional sources) [40]. We will examine points of divergence to better understand the relationship between theory and outcomes, find out how an intervention may work or not in particular conditions, and identify areas of opportunity for future research on how interventions could be improved.

## Results

The trial was launched in May 2017 and stopped in August 2017. At the time of submission of this article, data cleaning and analysis have not yet begun. Recruitment for the embedded process evaluation has paused with 11 physicians having completed semistructured interviews, as saturation of themes seemed to have been reached according to preliminary impressions formed during the interviews.

## Discussion

Our partnership with CCO allows for a province-wide trial of BCTs operationalized within emails that will simultaneously address applied questions of relevance to the organization and explore issues of interest to the broader implementation science literature. As such, it is an example of what we have previously described as an implementation science laboratory [41]. This kind of partnership enables low-cost trials by leveraging routinely collected administrative data, while improving research translation by ensuring that findings can be implemented in a sustainable way to improve health service performance [42]. This project will support CCO in its aim to ensure the continuous improvement and delivery of care to patients by “implementing provincial cancer prevention and screening programs, conducting research, and rapidly transferring knowledge of new research into improvements and innovations in clinical practice and cancer service delivery” [43]. From a scientific perspective, the lessons learned may provide insights regarding how to stimulate primary care physician engagement with audit and feedback tools, which may be generalizable to other contexts in Ontario and to other health care systems.

This work is not without limitations. First, generalizations drawn from focus group data are known to be problematic in that they are not usually indicative of participants’ individual opinions [44]. It could have been beneficial to get feedback from individual family physicians in a nongroup setting away from group of peers (eg, online survey, informal interviews) to verify that this matches the takeaways from the focus group. However, in our case, because we were using methods of cocreation, a group activity was required. More discussion relative to the intervention design phase will be reported further by our team. Second, inferences made from interviews in the qualitative process evaluation could be subject to a potential selection bias or volunteer bias. It is hard to recruit family physicians and therefore the ones agreeing to participate could have a specific pattern regarding SAR use and opinions. For example, they could be physicians who may be performing better and/or are more invested in understanding and improving their practice. For this reason, our interview findings may also lack generalizability to other family physicians—for example, the ones performing lower regarding cancer screening or/and less using the SAR.

The experiment also has limitations. Historically, according to internal CCO data, half of physicians receiving the SAR notification emails do not even open them. This suggests that features of the email such as subject line and sender may be important predictors of the user behavior we will be using as our primary and secondary outcomes. In our design phase, we worked with users and nonusers to choose sender and subject lines that we expect will optimize opening rates across all experimental conditions. It is possible that the sender or subject line may interact with our independent variables (BCTs) but the design of our trial does not allow us to identify such interactions. We plan to explore any potential such interaction in the process evaluation and anticipate that new hypotheses will be generated for testing in future trials. In addition, while we can assess whether emails were opened, we cannot know if emails were filtered to spam folders (in which case content redesign is unlikely to help). Finally, other physician sociodemographics such as ethnicity may also have been important covariates to examine but those data were not available at CCO.

A key strength of this study is its opportunistic and pragmatic design, which is likely to enhance external validity. We anticipate that the lessons learned may be applicable not only for CCO but for other health system organizations that need to support physicians in engaging with available tools to provide high-quality care.

## Appendix

Multimedia Appendix 1 Current version of the monthly email sent by Cancer Care Ontario.

Multimedia Appendix 2 Rewritten email containing the 3 behavior change techniques.

Multimedia Appendix 3 The 8 email interventions.

Multimedia Appendix 4 Semistructured interview guide.

## Abbreviations

BCT: behavior change technique
CCO: Cancer Care Ontario
FOBT: fecal occult blood test
LHIN: Local Health Integration Network
MOST: Multiphase Optimization Strategy
PEM: physician enrollment model
SAR: Screening Activity Report

## References

[1] Zapka JG, Lemon SC (2004). Interventions for patients, providers, and health care organizations. Cancer.

[2] Finney Rutten LJ, Nelson DE, Meissner HI (2004). Examination of population-wide trends in barriers to cancer screening from a diffusion of innovation perspective (1987-2000). Prev Med.

[3] Maxwell AE, Bastani R, Crespi CM, Danao LL, Cayetano RT (2011). Behavioral mediators of colorectal cancer screening in a randomized controlled intervention trial. Prev Med.

[4] Hanson K, Montgomery P, Bakker D, Conlon M (2009). Factors influencing mammography participation in Canada: an integrative review of the literature. Curr Oncol.

[5] Hewitson P, Ward AM, Heneghan C, Halloran SP, Mant D (2011). Primary care endorsement letter and a patient leaflet to improve participation in colorectal cancer screening: results of a factorial randomised trial. Br J Cancer.

[6] Ivers N, Jamtvedt G, Flottorp S, Young JM, Odgaard-Jensen J, French SD, O'Brien MA, Johansen M, Grimshaw J, Oxman AD (2012). Audit and feedback: effects on professional practice and healthcare outcomes. Cochrane Database Syst Rev.

[7] Arditi C, Rège-Walther M, Wyatt JC, Durieux P, Burnand B (2012). Computer-generated reminders delivered on paper to healthcare professionals: effects on professional practice and health care outcomes. Cochrane Database Syst Rev.

[8] Scott A, Sivey P, Ait Ouakrim D, Willenberg L, Naccarella L, Furler J, Young D (2011). The effect of financial incentives on the quality of health care provided by primary care physicians. Cochrane Database Syst Rev.

[9] Taitz JM, Lee TH, Sequist TD (2012). A framework for engaging physicians in quality and safety. BMJ Qual Saf.

[10] Ivers N, Barnsley J, Upshur R, Tu K, Shah B, Grimshaw J, Zwarenstein M (2014). “My approach to this job is...one person at a time”: Perceived discordance between population-level quality targets and patient-centred care. Can Fam Physician.

[11] van der Veer SN, de Keizer NF, Ravelli ACJ, Tenkink S, Jager KJ (2010). Improving quality of care. A systematic review on how medical registries provide information feedback to health care providers. Int J Med Inform.

[12] Ferlay J, Soerjomataram I, Dikshit R, Eser S, Mathers C, Rebelo M, Parkin DM, Forman D, Bray F (2015). Cancer incidence and mortality worldwide: sources, methods and major patterns in GLOBOCAN 2012. Int J Cancer.

[13] Fitzmaurice C, Dicker D, Pain A, Hamavid H, Moradi-Lakeh M, MacIntyre MF, Allen C, Hansen G, Woodbrook R, Wolfe C, Hamadeh RR, Moore A, Werdecker A, Gessner BD, Te Ao B, McMahon B, Karimkhani C, Yu C, Cooke GS, Schwebel DC, Carpenter DO, Pereira DM, Nash D, Kazi DS, De Diego L, Plass D, Ukwaja KN, Thurston GD, Yun Jin K, Simard EP, Mills E, Park E-K, Catalá-López F, deVeber G, Gotay C, Khan G, Hosgood D, Santos IS, Leasher JL, Singh J, Leigh J, Jonas JB, Jonas J, Sanabria J, Beardsley J, Jacobsen KH, Takahashi K, Franklin RC, Ronfani L, Montico M, Naldi L, Tonelli M, Geleijnse J, Petzold M, Shrime MG, Younis M, Yonemoto N, Breitborde N, Yip P, Pourmalek F, Lotufo PA, Esteghamati A, Hankey GJ, Ali R, Lunevicius R, Malekzadeh R, Dellavalle R, Weintraub R, Lucas R, Hay R, Rojas-Rueda D, Westerman R, Sepanlou SG, Nolte S, Patten S, Weichenthal S, Abera Semaw Ferede, Fereshtehnejad S-M, Shiue I, Driscoll T, Vasankari T, Alsharif U, Rahimi-Movaghar V, Vlassov VV, Marcenes WS, Mekonnen W, Melaku YA, Yano Y, Artaman A, Campos I, MacLachlan J, Mueller U, Kim D, Trillini M, Eshrati B, Williams HC, Shibuya K, Dandona R, Murthy K, Cowie B, Amare AT, Antonio CA, Castañeda-Orjuela C, van Gool CH, Violante F, Oh I-H, Deribe K, Soreide K, Knibbs L, Kereselidze M, Green M, Cardenas R, Roy N, Tillmann T, Tillman T, Li Y, Krueger H, Monasta L, Dey S, Sheikhbahaei S, Hafezi-Nejad N, Kumar GA, Sreeramareddy CT, Dandona L, Wang H, Vollset SE, Mokdad A, Salomon JA, Lozano R, Vos T, Forouzanfar M, Lopez A, Murray C, Naghavi M, Global Burden of Disease Cancer Collaboration (2015). The Global Burden of Cancer 2013. JAMA Oncol.

[14] (2014). The guide to clinical preventive services: recommendation of the US Preventive Service Task Force.

[15] Curry S, Byers T, Hewitt M (2003). Fulfilling the potential for cancer prevention and early detection.

[16] (2005). IARC Handbook of Cancer Prevention—Cervix Cancer Screening, Volume 10.

[17] (2017). Cancer Care Ontario.

[18] Cancer Quality Council of Ontario.

[19] Jonah L, Pefoyo AK, Lee A, Hader J, Strasberg S, Kupets R, Chiarelli AM, Tinmouth J (2017). Evaluation of the effect of an audit and feedback reporting tool on screening participation: the Primary Care Screening Activity Report (PCSAR). Prev Med.

[20] Collins LM, Kugler KC, Gwadz MV (2016). Optimization of multicomponent behavioral and biobehavioral interventions for the prevention and treatment of HIV/AIDS. AIDS Behav.

[21] Michie S, Atkins L, West R (2014). The Behaviour Change Wheel: A Guide to Designing Interventions.

[22] Michie S, Richardson M, Johnston M, Abraham C, Francis J, Hardeman W, Eccles MP, Cane J, Wood CE (2013). The Behavior Change Technique Taxonomy (v1) of 93 hierarchically clustered techniques: building an international consensus for the reporting of behavior change interventions. Ann Behav Med.

[23] Michie S, Johnston M, Abraham C, Lawton R, Parker D, Walker A (2005). Making psychological theory useful for implementing evidence based practice: a consensus approach. Qual Saf Health Care.

[24] Michie S, Johnston M, Francis J, Hardeman W, Eccles M (2008). From theory to intervention: mapping theoretically derived behavioural determinants to behaviour change techniques. Appl Psychol.

[25] Wyrick DL, Rulison KL, Fearnow-Kenney M, Milroy JJ, Collins LM (2014). Moving beyond the treatment package approach to developing behavioral interventions: addressing questions that arose during an application of the Multiphase Optimization Strategy (MOST). Transl Behav Med.

[26] Collins LM, Dziak JJ, Kugler KC, Trail JB (2014). Factorial experiments: efficient tools for evaluation of intervention components. Am J Prev Med.

[27] Nair V, Strecher V, Fagerlin A, Ubel P, Resnicow K, Murphy S, Little R, Chakraborty B, Zhang A (2008). Screening experiments and the use of fractional factorial designs in behavioral intervention research. Am J Public Health.

[28] Korn EL, Freidlin B (2016). Non-factorial analyses of two-by-two factorial trial designs. Clin Trials.

[29] McAlister FA, Straus SE, Sackett DL, Altman DG (2003). Analysis and reporting of factorial trials: a systematic review. JAMA.

[30] Statistics Canada.

[31] Hutchison B, Glazier R (2013). Ontario's primary care reforms have transformed the local care landscape, but a plan is needed for ongoing improvement. Health Aff (Millwood).

[32] Richardson A (2001). Screening and the number needed to treat. J Med Screen.

[33] Richardson AK, Potter JD (2014). Screening for colorectal cancer and prostate cancer: challenges for New Zealand. N Z Med J.

[34] van Ravesteyn NT, Stout NK, Schechter CB, Heijnsdijk EAM, Alagoz O, Trentham-Dietz A, Mandelblatt JS, de Koning HJ (2015). Benefits and harms of mammography screening after age 74 years: model estimates of overdiagnosis. J Natl Cancer Inst.

[35] Bulliard J-L, Levi F (2012). Mammography screening: time to reevaluate its impact?. Eur J Cancer Prev.

[36] Moore GF, Audrey S, Barker M, Bond L, Bonell C, Hardeman W, Moore L, O'Cathain A, Tinati T, Wight D, Baird J (2015). Process evaluation of complex interventions: Medical Research Council guidance. BMJ.

[37] Gale NK, Heath G, Cameron E, Rashid S, Redwood S (2013). Using the framework method for the analysis of qualitative data in multi-disciplinary health research. BMC Med Res Methodol.

[38] O'Cathain A, Murphy E, Nicholl J (2010). Three techniques for integrating data in mixed methods studies. BMJ.

[39] Farmer T, Robinson K, Elliott SJ, Eyles J (2006). Developing and implementing a triangulation protocol for qualitative health research. Qual Health Res.

[40] Lorencatto F, Gould NJ, McIntyre SA, During C, Bird J, Walwyn R, Cicero R, Glidewell L, Hartley S, Stanworth SJ, Foy R, Grimshaw JM, Michie S, Francis JJ, AFFINITIE programme (2016). A multidimensional approach to assessing intervention fidelity in a process evaluation of audit and feedback interventions to reduce unnecessary blood transfusions: a study protocol. Implement Sci.

[41] Ivers NM, Grimshaw JM (2016). Reducing research waste with implementation laboratories. Lancet.

[42] Wolfenden L, Yoong SL, Williams C, Grimshaw J, Durrheim DN, Gillham K, Wiggers J (2017). Embedding researchers in health service organizations improves research translation and health service performance: the Australian Hunter New England Population Health example. J Clin Epidemiol.

[43] (2015). Cancer Care Ontario.

[44] Sim J (1998). Collecting and analysing qualitative data: issues raised by the focus group. J Adv Nurs.

