# Supplementary figures and images for: Testing Behavior Change Techniques to Encourage Primary Care Physicians to Access Cancer Screening Audit and Feedback Reports: Protocol for a Factorial Randomized Experiment of Email Content
Source: JMIR Res Protoc. 2018 Feb 16;7(2):e11. doi: 10.2196/resprot.9090 (PMC5834752; doi:10.2196/resprot.9090)

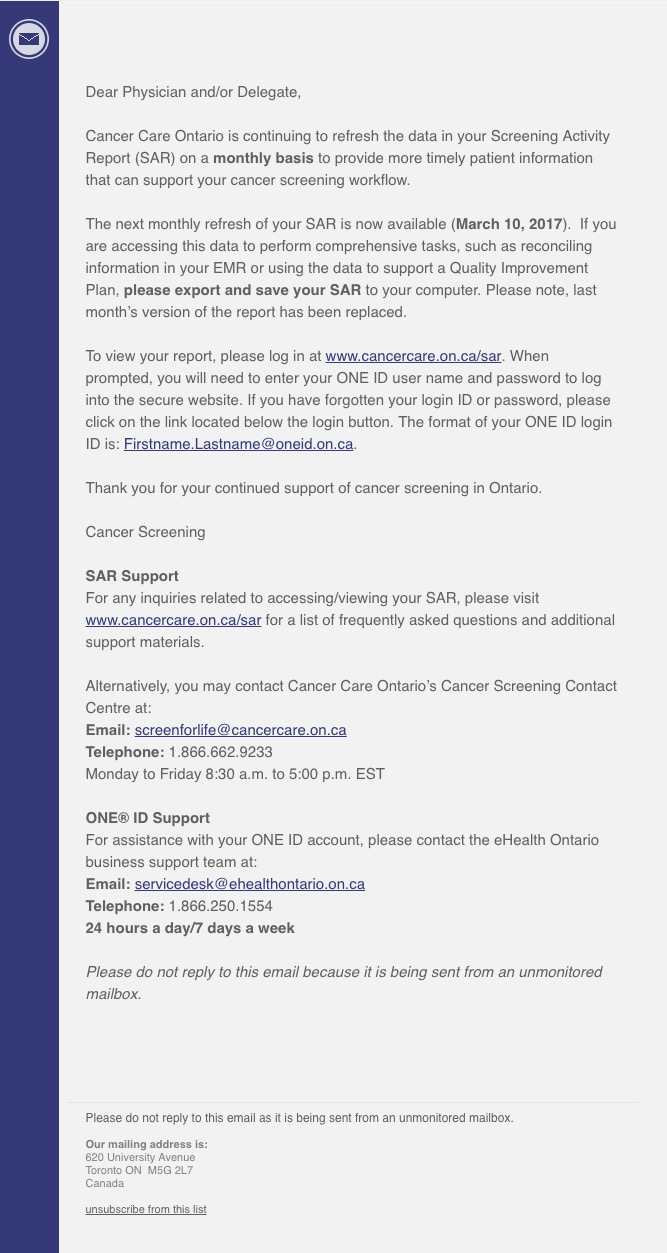

Supplement: Multimedia Appendix 1 [file resprot_v7i2e11_app1.jpg]

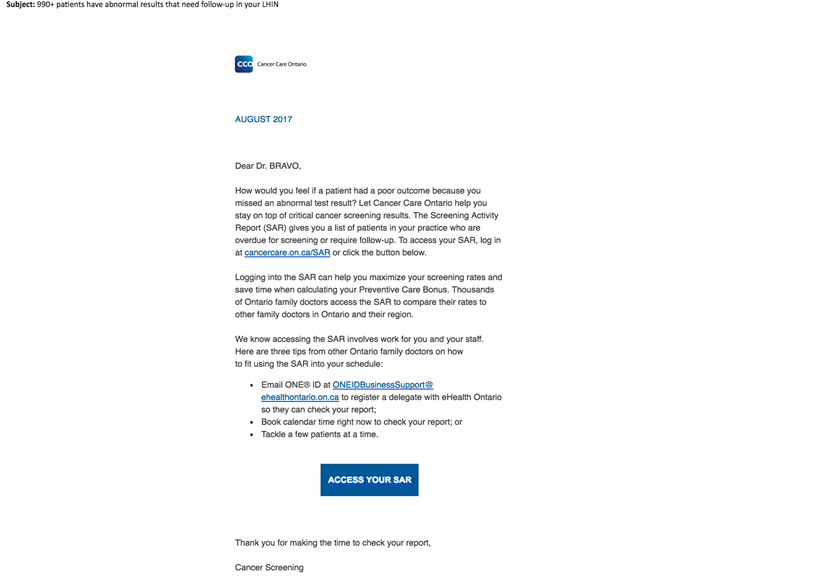

Supplement: Multimedia Appendix 2 [file resprot_v7i2e11_app2.png]
