# Supplementary material for: Testing Behavior Change Techniques to Encourage Primary Care Physicians to Access Cancer Screening Audit and Feedback Reports: Protocol for a Factorial Randomized Experiment of Email Content
Source: JMIR Res Protoc. 2018 Feb 16;7(2):e11. doi: 10.2196/resprot.9090 (PMC5834752; doi:10.2196/resprot.9090)
Supplement: Multimedia Appendix 4 [file resprot_v7i2e11_app4.pdf]

Multimedia Appendix 4: Semi-structured interview guide

<u>Introduction</u>

- Address terms of confidentiality: Consent forms, signature and recording device.
- Honorarium and receipts.

Explain the purpose and format of the interview, including duration

INTRODUCTION QUESTIONS

1. The goal of this interview is to better understand how the Cancer Care Ontario SAR monthly email prompts physicians to access their SAR, which requires an understanding of how physicians use their email. Can you describe how and when you usually check your email.

    <u>Prompts</u>: On a smartphone, on a computer, at home, at your office …

    <u>Follow-up</u>: SAR emails are usually sent during business hours on the 10th day of each month. How does this timing influence your ability to focus on incoming emails?

    <u>What would be the ideal time for you to receive an email reminder?</u>

Let's talk specifically about the Screening Activity Report email reminder you received around May 10th/June 10th/July 10th/August 10th 2017 from Cancer Care Ontario (Ask them to have it in front of them).

<u>Notes:</u> *Ask if they'd agree to send it to us (to know what their exposure was).*

If they don't have it, we'll send them a fully turned ON version. "We'll send you a template we used. It may be different from the one you received originally but will be useful to our discussion. We'll take 5 minutes to read it just in one moment".

Take 5 minutes to read the email we've just sent you.

2. Does this email or any part of it look familiar to you?

    IF **YES**, What looks familiar about it?

    Do you remember where you were when you received this email? *(Draw their attention to when it was sent using the time stamp)*

    IF **NO**, Can you talk through what you're thinking when you read this email?

    Probe around subject line and 'active' content.

3. Think back to your thought process when opening this email, if possible. If you don't remember, focus on your current thought process. Can you talk me through what you're paying attention to (or not) and what you are thinking?

    <u>Prompts</u>: Ideally reaction to the content. Feedback on the subject line, from…, how they sort emails: by subject line, from…

IMPACT & BEHAVIOUR CHANGE TECHNIQUES

4. Did this email prompt you to check your SAR, or to schedule time to do so in the future?

    Follow-up: Can you walk me through what part of the email triggered that response and how?

5. How or why do you feel the email influenced (or failed to influence) your cancer screening practices?
   <u>*Follow-up*</u> *[if changes reported]:* What about the email encouraged that change?
   <u>*Follow-up*</u> *[if no changes reported]:* What could the email have included to encourage action?

PROBLEM SOLVING

6. What do you think about the strategies listed in the revised email?

7. Did the strategies change the way you think about how or when you access the SAR?

8. What are strategies you use personally or in your practice that help remind you to check your SAR?
   Note: to know if the email only acts as a reminder or as a motivator.

ANTICIPATED REGRET

9. The first statement of the email says…*How would you feel if a patient had a poor outcome because you missed an abnormal test result?* What was your immediate reaction to that statement? What went through your mind when you read that?

10. Did this statement change the way you think about cancer screening?
    If <u>**yes**</u>, what did you notice change?

11. Physicians are often motivated by achieving good outcomes for their patients. What types of scenarios related to cancer screening in primary care would you want to be notified of to avoid potential regret?
    Follow-up: Which of those scenarios are the highest priority for you?

MATERIAL INCENTIVE

12. The email links the use of the SAR report to your prevention screening bonus. How does the knowledge of this incentive impact your approach to cancer screening?

13. Are there additional incentives that motivate you to access your SAR?

14. How do current incentives motivate you to adhere to cancer screening guidelines (i.e. Preventive care bonus, CME credits)

DID NOT ACCESS TO THE SAR BETWEEN MAY AND AUGUST 2017

15. What do you think the email could have said to have motivated you to access your SAR?
16. If we could do one thing better to encourage primary care physicians like yourself to access your SAR, what might that be?
