# Supplementary material for: Testing Behavior Change Techniques to Encourage Primary Care Physicians to Access Cancer Screening Audit and Feedback Reports: Protocol for a Factorial Randomized Experiment of Email Content
Source: JMIR Res Protoc. 2018 Feb 16;7(2):e11. doi: 10.2196/resprot.9090 (PMC5834752; doi:10.2196/resprot.9090)
Supplement: Multimedia Appendix 3 [file resprot_v7i2e11_app3.pdf]

Multimedia Appendix 3: The 8 email interventions

Email 1 (87 words, 429 characters, Readability: Passive sentences =0%, Flesch Reading Ease = 48.4, Flesch-Kincaid Grade Level = 11.2): No factors

Subject: 990+ patients have abnormal results that need follow-up in your LHIN

CCO Cancer Care Ontario

AUGUST 2017

Dear Dr. [PHYSICIAN LAST NAME],

Let Cancer Care Ontario help you stay on top of critical cancer screening results. The Screening Activity Report (SAR) gives you a list of patients in your practice who are overdue for screening or require follow-up. To access your SAR, log in at cancercare.on.ca/SAR or click the button below.

Thousands of Ontario family doctors access the SAR to compare their rates to other family doctors in Ontario and their region.

ACCESS YOUR SAR

Thank you for making the time to check your report,

Cancer Screening

Forgot your ONE® ID login or password? Reset it at
https://oneid.ehealthontario.ca/forgotPassword

ONE® ID Support

For assistance with your ONE® ID account, please contact eHealth Ontario at:

Email: servicedesk@ehealthontario.on.ca

Telephone: 1.866.250.1554, 24 hours a day/7 days a week

Need more help?

Email: screenforlife@cancercare.on.ca

Telephone: 1.866.662.9233, Monday to Friday 8:30 a.m. to 5 p.m. EST

Watch: The online SAR tutorial

Copyright © 2017 Cancer Care Ontario, All rights reserved

You are receiving this email because you are registered with Cancer Care Ontario to receive screening reports containing your patients' data. Because of privacy laws, we cannot email you your report; you must log in to view it online. Thank you for your understanding.

You may notice that this email is different from your previous SAR emails. We are working with researchers to test ways to improve our communication with you. If you have questions about this research please contact us at sarproject@wchospital.ca.

Please do not reply to this email as it is being sent from an unmonitored mailbox.

Our mailing address is:
Cancer Care Ontario
620 University Ave,
Toronto, On M5G L27
Canada

Add us to your address book

Want to change how you receive these emails?
You can update your preferences or unsubscribe from this list

Email 2 (182 words, 688 characters, Readability: Passive sentences =0%, Flesch Reading Ease = 53.6, Flesch-Kincaid Grade Level = 10.4): Anticipated Regret

Subject: 990+ patients have abnormal results that need follow-up in your LHIN

Cancer Care Ontario

AUGUST 2017

Dear Dr. [PHYSICIAN LAST NAME],

How would you feel if a patient had a poor outcome because you missed an abnormal test result? Let Cancer Care Ontario help you stay on top of critical cancer screening results. The Screening Activity Report (SAR) gives you a list of patients in your practice who are overdue for screening or require follow-up. To access your SAR, log in at cancercare.on.ca/SAR or click the button below.

Thousands of Ontario family doctors access the SAR to compare their rates to other family doctors in Ontario and their region.

ACCESS YOUR SAR

Thank you for making the time to check your report,

Cancer Screening

Forgot your ONE® ID login or password? Reset it at

https://oneid.ehealthontario.ca/forgotPassword

ONE® ID Support

For assistance with your ONE® ID account, please contact eHealth Ontario at:

Email: servicedesk@ehealthontario.on.ca

Telephone: 1.866.250.1554, 24 hours a day/7 days a week

Need more help?

Email: screenforlife@cancercare.on.ca

Telephone: 1.866.662.9233, Monday to Friday 8:30 a.m. to 5 p.m. EST

Watch: The online SAR tutorial

Copyright © 2017 Cancer Care Ontario, All rights reserved

You are receiving this email because you are registered with Cancer Care Ontario to receive screening reports containing your patients' data. Because of privacy laws, we cannot email you your report; you must log in to view it online. Thank you for your understanding.

You may notice that this email is different from your previous SAR emails. We are working with researchers to test ways to improve our communication with you. If you have questions about this research please contact us at sarproject@wchospital.ca.

Please do not reply to this email as it is being sent from an unmonitored mailbox.

Our mailing address is:
Cancer Care Ontario
620 University Ave,
Toronto, On M5G L27
Canada

Add us to your address book

Want to change how you receive these emails?
You can update your preferences or unsubscribe from this list

Email 3 (107 words, 532 characters, Readability: Passive sentences =0%, Flesch Reading Ease = 49.8, Flesch-Kincaid Grade Level = 11.0): Material Incentive (behaviour)

Subject: 990+ patients have abnormal results that need follow-up in your LHIN

CCO Cancer Care Ontario

AUGUST 2017

Dear Dr. [PHYSICIAN LAST NAME],

Let Cancer Care Ontario help you stay on top of critical cancer screening results. The Screening Activity Report (SAR) gives you a list of patients in your practice who are overdue for screening or require follow-up. To access your SAR, log in at cancercare.on.ca/SAR or click the button below.

Logging into the SAR can help you maximize your screening rates and save time when calculating your Preventive Care Bonus. Thousands of Ontario family doctors access the SAR to compare their rates to other family doctors in Ontario and their region.

ACCESS YOUR SAR

Thank you for making the time to check your report,

Cancer Screening

Forgot your ONE® ID login or password? Reset it at

https://oneid.ehealthontario.ca/forgotPassword

ONE® ID Support

For assistance with your ONE® ID account, please contact eHealth Ontario at:

Email: servicedesk@ehealthontario.on.ca

Telephone: 1.866.250.1554, 24 hours a day/7 days a week

Need more help?

Email: screenforlife@cancercare.on.ca

Telephone: 1.866.662.9233, Monday to Friday 8:30 a.m. to 5 p.m. EST

Watch: The online SAR tutorial

Copyright © 2017 Cancer Care Ontario, All rights reserved

You are receiving this email because you are registered with Cancer Care Ontario to receive screening reports containing your patients' data. Because of privacy laws, we cannot email you your report; you must log in to view it online. Thank you for your understanding.

You may notice that this email is different from your previous SAR emails. We are working with researchers to test ways to improve our communication with you. If you have questions about this research please contact us at sarproject@wchospital.ca.

Please do not reply to this email as it is being sent from an unmonitored mailbox.

Our mailing address is:
Cancer Care Ontario
620 University Ave,
Toronto, On M5G L27
Canada

Add us to your address book

Want to change how you receive these emails?
You can update your preferences or unsubscribe from this list

Email 4 (153 words, 753 characters, Readability: Passive sentences =0%, Flesch Reading Ease = 58.0, Flesch-Kincaid Grade Level = 9.0): Problem Solving

Subject: 990+ patients have abnormal results that need follow-up in your LHIN

Cancer Care Ontario

AUGUST 2017

Dear Dr. [PHYSICIAN LAST NAME],

Let Cancer Care Ontario help you stay on top of critical cancer screening results. The Screening Activity Report (SAR) gives you a list of patients in your practice who are overdue for screening or require follow-up. To access your SAR, log in at cancercare.on.ca/SAR or click the button below.

Thousands of Ontario family doctors access the SAR to compare their rates to other family doctors in Ontario and their region.

We know accessing the SAR involves work for you and your staff. Here are three tips from other Ontario family doctors on how to fit using the SAR into your schedule:

- Email ONE® ID at ONEIDBusinessSupport@ehealthontario.on.ca to register a delegate with eHealth Ontario so they can check your report;
- Book calendar time right now to check your report; or
- Tackle a few patients at a time.

ACCESS YOUR SAR

Thank you for making the time to check your report,

Cancer Screening

Forgot your ONE® ID login or password? Reset it at

https://oneid.ehealthontario.ca/forgotPassword

ONE® ID Support

For assistance with your ONE® ID account, please contact eHealth Ontario at:

Email: servicedesk@ehealthontario.on.ca

Telephone: 1.866.250.1554, 24 hours a day/7 days a week

Need more help?

Email: screenforlife@cancercare.on.ca

Telephone: 1.866.662.9233, Monday to Friday 8:30 a.m. to 5 p.m. EST

Watch: The online SAR tutorial

Copyright © 2017 Cancer Care Ontario, All rights reserved

You are receiving this email because you are registered with Cancer Care Ontario to receive screening reports containing your patients' data. Because of privacy laws, we cannot email you your report; you must log in to view it online. Thank you for your understanding.

You may notice that this email is different from your previous SAR emails. We are working with researchers to test ways to improve our communication with you. If you have questions about this research please contact us at sarproject@wchospital.ca.

Please do not reply to this email as it is being sent from an unmonitored mailbox.

Our mailing address is:
Cancer Care Ontario
620 University Ave,
Toronto, On M5G L27
Canada

Add us to your address book

Want to change how you receive these emails?
You can update your preferences or unsubscribe from this list

Email 5 (125 words, 609 characters, Readability: Passive sentences =0%, Flesch Reading Ease = 53.9, Flesch-Kincaid Grade Level = 10.4): Anticipated Regret + Material Incentive (behaviour)

Subject: 990+ patients have abnormal results that need follow-up in your LHIN

Cancer Care Ontario

AUGUST 2017

Dear Dr. [PHYSICIAN LAST NAME],

How would you feel if a patient had a poor outcome because you missed an abnormal test result? Let Cancer Care Ontario help you stay on top of critical cancer screening results. The Screening Activity Report (SAR) gives you a list of patients in your practice who are overdue for screening or require follow-up. To access your SAR, log in at cancercare.on.ca/SAR or click the button below.

Logging into the SAR can help you maximize your screening rates and save time when calculating your Preventive Care Bonus. Thousands of Ontario family doctors access the SAR to compare their rates to other family doctors in Ontario and their region.

ACCESS YOUR SAR

Thank you for making the time to check your report,

Cancer Screening

Forgot your ONE® ID login or password? Reset it at

https://oneid.ehealthontario.ca/forgotPassword

ONE® ID Support

For assistance with your ONE® ID account, please contact eHealth Ontario at:

Email: servicedesk@ehealthontario.on.ca

Telephone: 1.866.250.1554, 24 hours a day/7 days a week

Need more help?

Email: screenforlife@cancercare.on.ca

Telephone: 1.866.662.9233, Monday to Friday 8:30 a.m. to 5 p.m. EST

Watch: The online SAR tutorial

Copyright © 2017 Cancer Care Ontario, All rights reserved

You are receiving this email because you are registered with Cancer Care Ontario to receive screening reports containing your patients' data. Because of privacy laws, we cannot email you your report; you must log in to view it online. Thank you for your understanding.

You may notice that this email is different from your previous SAR emails. We are working with researchers to test ways to improve our communication with you. If you have questions about this research please contact us at sarproject@wchospital.ca.

Please do not reply to this email as it is being sent from an unmonitored mailbox.

Our mailing address is:
Cancer Care Ontario
620 University Ave,
Toronto, On M5G L27
Canada

Add us to your address book

Want to change how you receive these emails?
You can update your preferences or unsubscribe from this list

Email 6 (171 words, 830 characters, Readability: Passive sentences =0%, Flesch Reading Ease = 60.9, Flesch-Kincaid Grade Level = 8.7): Anticipated Regret + Problem Solving

Subject: 990+ patients have abnormal results that need follow-up in your LHIN

CCO Cancer Care Ontario

AUGUST 2017

Dear Dr. [PHYSICIAN LAST NAME],

How would you feel if a patient had a poor outcome because you missed an abnormal test result? Let Cancer Care Ontario help you stay on top of critical cancer screening results. The Screening Activity Report (SAR) gives you a list of patients in your practice who are overdue for screening or require follow-up. To access your SAR, log in at cancercare.on.ca/SAR or click the button below.

Thousands of Ontario family doctors access the SAR to compare their rates to other family doctors in Ontario and their region.

We know accessing the SAR involves work for you and your staff. Here are three tips from other Ontario family doctors on how to fit using the SAR into your schedule:

- Email ONE® ID at ONEIDBusinessSupport@ehealthontario.on.ca to register a delegate with eHealth Ontario so they can check your report;
- Book calendar time right now to check your report; or
- Tackle a few patients at a time.

ACCESS YOUR SAR

Thank you for making the time to check your report,

Cancer Screening

Forgot your ONE® ID login or password? Reset it at
https://oneid.ehealthontario.ca/forgotPassword

ONE® ID Support

For assistance with your ONE® ID account, please contact eHealth Ontario at:

Email: servicedesk@ehealthontario.on.ca

Telephone: 1.866.250.1554, 24 hours a day/7 days a week

Need more help?

Email: screenforlife@cancercare.on.ca

Telephone: 1.866.662.9233, Monday to Friday 8:30 a.m. to 5 p.m. EST

Watch: The online SAR tutorial

Copyright © 2017 Cancer Care Ontario, All rights reserved

You are receiving this email because you are registered with Cancer Care Ontario to receive screening reports containing your patients' data. Because of privacy laws, we cannot email you your report; you must log in to view it online. Thank you for your understanding.

You may notice that this email is different from your previous SAR emails. We are working with researchers to test ways to improve our communication with you. If you have questions about this research please contact us at sarproject@wchospital.ca.

Please do not reply to this email as it is being sent from an unmonitored mailbox.

Our mailing address is:
Cancer Care Ontario
620 University Ave,
Toronto, On M5G L27
Canada

Add us to your address book

Want to change how you receive these emails?
You can update your preferences or unsubscribe from this list

Email 7 (173 words, 856 characters, Readability: Passive sentences =0%, Flesch Reading Ease = 57.7, Flesch-Kincaid Grade Level = 9.2): Material Incentive (behaviour) + Problem Solving

Subject: 990+ patients have abnormal results that need follow-up in your LHIN

Cancer Care Ontario

AUGUST 2017

Dear Dr. [PHYSICIAN LAST NAME],

Let Cancer Care Ontario help you stay on top of critical cancer screening results. The Screening Activity Report (SAR) gives you a list of patients in your practice who are overdue for screening or require follow-up. To access your SAR, log in at cancercare.on.ca/SAR or click the button below.

Logging into the SAR can help you maximize your screening rates and save time when calculating your Preventive Care Bonus. Thousands of Ontario family doctors access the SAR to compare their rates to other family doctors in Ontario and their region.

We know accessing the SAR involves work for you and your staff. Here are three tips from other Ontario family doctors on how to fit using the SAR into your schedule:

- Email ONE® ID at ONEIDBusinessSupport@ehealthontario.on.ca to register a delegate with eHealth Ontario so they can check your report;
- Book calendar time right now to check your report; or
- Tackle a few patients at a time.

ACCESS YOUR SAR

Thank you for making the time to check your report,

Cancer Screening

Forgot your ONE® ID login or password? Reset it at
https://oneid.ehealthontario.ca/forgotPassword

ONE® ID Support

For assistance with your ONE® ID account, please contact eHealth Ontario at:

Email: servicedesk@ehealthontario.on.ca

Telephone: 1.866.250.1554, 24 hours a day/7 days a week

Need more help?

Email: screenforlife@cancercare.on.ca

Telephone: 1.866.662.9233, Monday to Friday 8:30 a.m. to 5 p.m. EST

Watch: The online SAR tutorial

Copyright © 2017 Cancer Care Ontario, All rights reserved

You are receiving this email because you are registered with Cancer Care Ontario to receive screening reports containing your patients' data. Because of privacy laws, we cannot email you your report; you must log in to view it online. Thank you for your understanding.

You may notice that this email is different from your previous SAR emails. We are working with researchers to test ways to improve our communication with you. If you have questions about this research please contact us at sarproject@wchospital.ca.

Please do not reply to this email as it is being sent from an unmonitored mailbox.

Our mailing address is:
Cancer Care Ontario
620 University Ave,
Toronto, On M5G L27
Canada

Add us to your address book

Want to change how you receive these emails?
You can update your preferences or unsubscribe from this list

Email 8 (191 words, 933 characters, Readability: Passive sentences =0%, Flesch Reading Ease = 60.1, Flesch-Kincaid Grade Level = 8.9): Anticipated Regret + Material Incentive (behaviour) + Problem Solving

Subject: 990+ patients have abnormal results that need follow-up in your LHIN

CCO Cancer Care Ontario

AUGUST 2017

Dear Dr. [PHYSICIAN LAST NAME],

How would you feel if a patient had a poor outcome because you missed an abnormal test result? Let Cancer Care Ontario help you stay on top of critical cancer screening results. The Screening Activity Report (SAR) gives you a list of patients in your practice who are overdue for screening or require follow-up. To access your SAR, log in at cancercare.on.ca/SAR or click the button below.

Logging into the SAR can help you maximize your screening rates and save time when calculating your Preventive Care Bonus. Thousands of Ontario family doctors access the SAR to compare their rates to other family doctors in Ontario and their region.

We know accessing the SAR involves work for you and your staff. Here are three tips from other Ontario family doctors on how to fit using the SAR into your schedule:

- Email ONE® ID at ONEIDBusinessSupport@ehealthontario.on.ca to register a delegate with eHealth Ontario so they can check your report;
- Book calendar time right now to check your report; or
- Tackle a few patients at a time.

ACCESS YOUR SAR

Thank you for making the time to check your report,

Cancer Screening

Forgot your ONE® ID login or password? Reset it at
https://oneid.ehealthontario.ca/forgotPassword

ONE® ID Support

For assistance with your ONE® ID account, please contact eHealth Ontario at:

Email: servicedesk@ehealthontario.on.ca

Telephone: 1.866.250.1554, 24 hours a day/7 days a week

Need more help?

Email: screenforlife@cancercare.on.ca

Telephone: 1.866.662.9233, Monday to Friday 8:30 a.m. to 5 p.m. EST

Watch: The online SAR tutorial

Copyright © 2017 Cancer Care Ontario, All rights reserved

You are receiving this email because you are registered with Cancer Care Ontario to receive screening reports containing your patients' data. Because of privacy laws, we cannot email you your report; you must log in to view it online. Thank you for your understanding.

You may notice that this email is different from your previous SAR emails. We are working with researchers to test ways to improve our communication with you. If you have questions about this research please contact us at sarproject@wchospital.ca.

Please do not reply to this email as it is being sent from an unmonitored mailbox.

Our mailing address is:
Cancer Care Ontario
620 University Ave,
Toronto, On M5G L27
Canada

Add us to your address book

Want to change how you receive these emails?
You can update your preferences or unsubscribe from this list
